# Mapping the trajectories for women and their babies from births planned at home, in a birth centre or in a hospital in New South Wales, Australia, between 2000 and 2012

**DOI:** 10.1186/s12884-019-2584-0

**Published:** 2019-12-21

**Authors:** Vanessa L. Scarf, Rosalie Viney, Serena Yu, Maralyn Foureur, Chris Rossiter, Hannah Dahlen, Charlene Thornton, Seong Leang Cheah, Caroline S. E. Homer

**Affiliations:** 1 Centre for Midwifery, Child and Family Health, University of Technology Sydney, Sydney, New South Wales Australia; 2 Centre for Health Economic Research and Evaluation (CHERE), University of Technology Sydney, PO Box 123, Broadway, Ultimo, NSW 2007 Australia; 3 School of Nursing and Midwifery, Western Sydney University, Sydney, Australia; 4 College of Nursing and Health Sciences, Flinders University, Adelaide, Australia

**Keywords:** Home birth, Birth centre, Place of birth, Midwife, Transfer

## Abstract

**Background:**

In New South Wales (NSW) Australia, women at low risk of complications can choose from three birth settings: home, birth centre and hospital. Between 2000 and 2012, around 6.4% of pregnant women planned to give birth in a birth centre (6%) or at home (0.4%) and 93.6% of women planned to birth in a hospital. A proportion of the woman in the home and birth centre groups transferred to hospital. However, their pathways or trajectories are largely unknown.

**Aim:**

The aim was to map the trajectories and interventions experienced by women and their babies from births planned at home, in a birth centre or in a hospital over a 13-year period in NSW.

**Methods:**

Using population-based linked datasets from NSW, women at low risk of complications, with singleton pregnancies, gestation 37–41 completed weeks and spontaneous onset of labour were included. We used a decision tree framework to depict the trajectories of these women and estimate the probabilities of the following: giving birth in their planned setting; being transferred; requiring interventions and neonatal admission to higher level hospital care. The trajectories were analysed by parity.

**Results:**

Over a 13-year period, 23% of nulliparous and 0.8% of multiparous women planning a home birth were transferred to hospital. In the birth centre group, 34% of nulliparae and 12% of multiparas were transferred to a hospital. Normal vaginal birth rates were higher in multiparous women compared to nulliparous women in all settings. Neonatal admission to SCN/NICU was highest in the planned hospital group for nulliparous women (10.1%), 7.1% for nulliparous women planning a birth centre birth and 5.1% of nulliparous women planning a homebirth. Multiparas had lower admissions to SCN/NICU for all thee settings (hospital 6.3%, BC 3.6%, home 1.6%, respectively).

**Conclusions:**

Women who plan to give birth at home or in a birth centre have high rates of vaginal birth, even when transferred to hospital. Evidence on the trajectories of women who choose to give birth at home or in birth centres will assist the planning, costing and expansion of models of care in NSW.

## Background

In Australia, as in many high-income countries, women can choose to give birth at home, in a birth centre or in a birth unit. In New South Wales (NSW), the most populous state in Australia, there are over 97,000 births a year [1]. Annual figures from the most recent data (2016) show that in this state, 96.6% of women gave birth in a hospital labour ward, 2.2% gave birth in a birth centre and 0.2% gave birth at home [1].

There is now strong evidence that for women with a healthy pregnancy, especially those having their second or subsequent baby, giving birth at home or in a BC is a safe option [2–12]. The small proportion of women who have used BCs in NSW in 2016 (2.2%) or who have chosen to give birth at home (0.2%) reflects either the lack of availability or desirability of such services, notwithstanding the demand for greater choice of birth setting by women and health practitioners [13–15].

The Australian National Review of Maternity Services released in 2009 sought perspectives from a range of stakeholders regarding maternity services in Australia in order to inform priorities for the development of the National Maternity Services Plan (The Plan) which was released in 2011 [16]. As a result, The Plan outlined priorities including increasing access to local maternity care by expanding the range of models of care with an associated increase in birth setting options [16]. The Plan was a result of submissions from women who indicated they want options regarding their pregnancy care and choice of place of birth. During the Maternity Services Review, over 900 submissions were received, the vast majority (*n* = 832) were made by women and maternity care providers [17]. Consistent themes emerged such as wanting increased access to a midwife-led and continuity of care and more options for place of birth, including homebirth and birth centres [13, 14].

According to the 2016 NSW Mothers and Babies Report [1] there are 62 maternity hospitals with birth rates over 200 per year. This number comprises 47 public hospitals and 15 private hospitals. There are three possible settings in which to choose to give birth – in hospital, in a birth centre or at home in NSW, however these settings are not necessarily available across the state. A hospital labour ward (HLW) is within a hospital (public and private) and is staffed by midwives and doctors. There are five birth centres (BC) co-located within hospital grounds or adjacent to hospital labour wards, they are staffed by midwives (although obstetricians and registrars are available in some settings if interventions are required) and are designed to provide a home-like environment. There are also five free-standing midwifery led birth centres in NSW which are located within a hospital campus, albeit some distance from obstetric and neonatal specialties. Women who require transfer to higher level care at these birth centres are transported by car or ambulance to the closest maternity hospital.

### Birth trajectories

While women usually choose where they would like to give birth at the beginning of pregnancy, the process is dynamic due to complications or risk factors that may develop, making the pathway or trajectory for women who plan to give birth at home or in a birth centre difficult to predict at a service level. A woman intending a homebirth, for example, may commence her pregnancy with no significant history of illness or pathology only to find her plans changed as the pregnancy continues and a complication arises. This may result in a change of birth setting, either during the pregnancy or in labour; the latter made sometimes more difficult due to a lack of integration between the providers of homebirth and hospital services [18]. In countries where homebirth and freestanding birth centres are well integrated into maternity services (UK, Netherlands), transfers between places of birth are facilitated by local policies and protocols which support the need to change location, including during labour, to the preferred or more appropriate birth setting [19]. By contrast, a maternity system lacking in integration between providers and places of birth, as is common across Australia, creates barriers for a smooth transition from home to hospital where indicated [18].

Transfer rates from planned homebirth to hospital vary by country as well as by parity, with predictably lower rates in multiparous women. The rates of intrapartum transfer from home to hospital in studies over the past 10 years from a number of high-income countries varied from 8.8 to 21.0% overall [4, 9, 20–22]. When stratified by parity, the rates were 24 to 39.1% for nulliparous women and 4.8 to 12.3% for multiparous women. Transfer from a midwifery unit (either alongside or freestanding) to hospital were 12.4 to 33.9% overall [4, 9, 10, 22–25] and by parity, 25.4 to 37.8% for nulliparous women and 5.3 to 14.0% for multiparous women. Reasons for intrapartum transfer range from request for analgesia and slow progress in labour (non-urgent) to fetal distress and haemorrhage (urgent) the latter being less common [21, 24, 26, 27].

While transfer rates in NSW have been reported overall, little is known about what happens to women who commence labour in their planned place of birth, and their babies during and after transfer. Anecdotally, support for the expansion of homebirth and birth centre services has been hampered by a belief that this intrapartum change of venue adds a layer of unnecessary risk to women and their babies [28, 29]. This study explores these events during labour, which include planned place of birth, transfer from home or a birth centre to hospital, actual place of birth, mode of birth and neonatal admission to special care nursery/neonatal intensive care unit (SCN/NICU), described as birth trajectories, for a low-risk cohort of women from NSW from 2000 to 2012. This information will aid in our understanding of the intrapartum transfer rate and subsequent interventions and assist with maternity service development and expansion of options for women interested in birth at home or in a birth centre. It will also inform understanding of the costs in different settings, because the costs of birth at home or in a birth centre should include the cost associated with transfer where applicable.

The aim therefore was to investigate the birth trajectories of women at low risk of complications who at the end of pregnancy plan to give birth at home, in a birth centre or a hospital labour ward. The development of this decision tree framework was also undertaken to inform a future costing of these birth settings.

## Methods

### Design: decision tree modelling

**Fig. 1:**
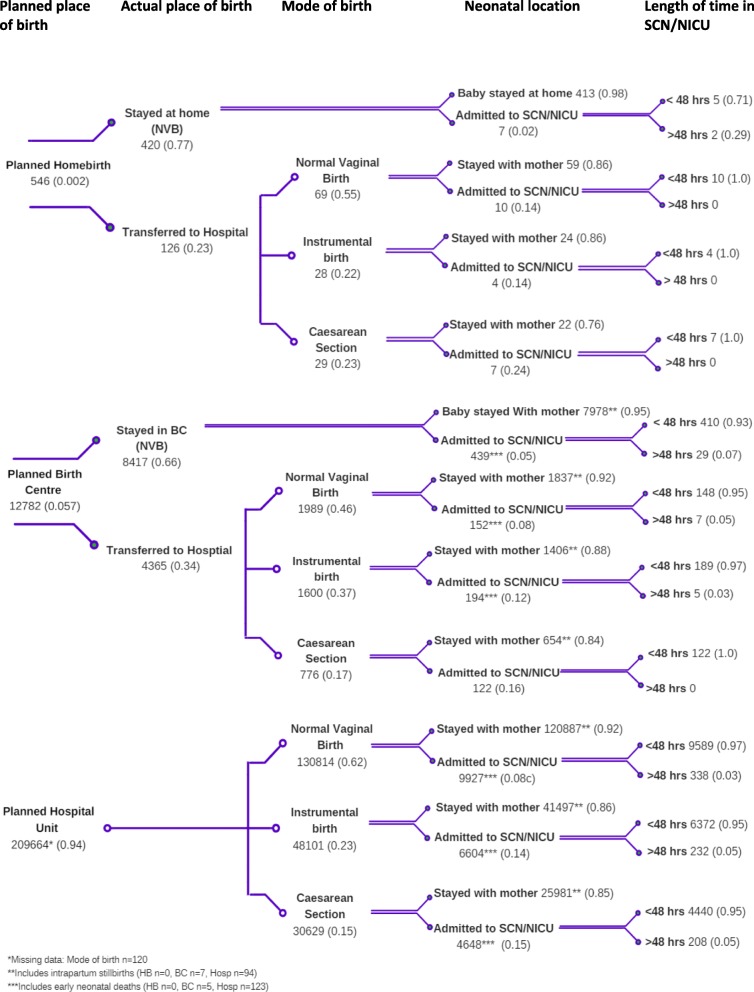
Nulliparous women at low risk of complications between 2000 and 2012 (*n* = 222,992)

A retrospective population-based cohort study using linked health data was undertaken. The study draws on the framework of decision analytic modelling to construct a decision tree. Typically, a decision tree model provides a simplified framework of decisions that are made at different points in a treatment schedule depending on outcomes or events at a given time, “under conditions of uncertainty” and are mutually exclusive [30, 31]. The decision tree developed for this study depicts the trajectories of women as their labour progressed by analysing linked health data, moving from their plans at the onset of labour to the birth of their child. We report probabilities at each ‘node’ of the decision tree, stratified by parity. We illustrated these trajectories in a decision tree (Fig. 1) with the events (branch) of the decision tree in Table 1.

**Table 1:** Description of decision branches

Decision Node	Branch 1 Intended place of birth	Branch 2 Intrapartum events	Branch 3 Mode of birth	Branch 4 Neonatal events	Branch 5 SCN/NICU
Planned place of birth at onset of labour by parity	Home Birth Centre Hospital Labour ward	Actual birth in planned setting or transfer to hospital labour ward	Normal Vaginal Birth (NVB) Instrumental birth – Forceps, vacuum (IB) Caesarean Section (CS)	Post-birth remains with mother in birth setting or Admission to special care nursery or Neonatal Intensive Care (SCN or NICU)	Admission to SCN/NICU < 48 h or > 48 h

A decision tree is interpreted left to right, on the left is the decision node representing the planned place of birth at the onset of labour for women with a healthy pregnancy at low risk of complications. The pathways or trajectories represent the events that occurred for these women and their infants and are defined at each ‘chance’ node moving right, from which a branch emanates. The alternative trajectories are mutually exclusive and the probability of each branch is calculated. While decision tree analysis is used for modelling options in terms of end-points and costs, we are using the framework to depict and quantify the trajectories of women by their planned birth setting. We populated the decision tree in this study with data analysed from a linked population-based data set obtained from NSW Ministry of Health.

### Setting

This study investigates the trajectories of women in New South Wales who planned to give birth in the birth settings described above. During the study period (2000–2012), there were six alongside BCs and three freestanding BCs in NSW. Freestanding BCs accounted for approximately 15% of BC births between 2000 and 2012. The data did not permit separate analysis by type of BC.

The number of maternity hospitals in NSW has remained constant over the period. The majority of homebirth services were and still are provided by midwives in private practice who are employed directly by women. There are a small number of publicly funded homebirth services which are staffed by midwives employed by public hospitals [32, 33].

### Data sources

Data for all women who gave birth in NSW between January 2000 and December 2012 and all babies born between January 2000 and December 2012 of greater than 400 g and 20 weeks gestation were included. Four datasets were linked:

1. NSW Perinatal Data Collection (PDC): Midwives and doctors collect data routinely on all women who give birth in NSW, at the point of care, most often through electronic medical record platforms. Maternal and infant data is collected on all births greater than 20 weeks gestation or 400 g birthweight.
2. NSW Admitted Patient Data Collection (APDC): This is a record of all NSW hospital inpatient services including public and private hospitals, public psychiatric hospitals, and private day procedure centres. Clinical data is recorded using the International Classification of Diseases- Australian Modification (ICD-AM) codes.
3. NSW Registry of Births, Deaths and Marriages (NSWRBDM): Data on all registered births and deaths.
4. Australian Bureau of Statistics (ABS) mortality data including primary cause and date of death.

### Sample and inclusion criteria

The cohort was derived from the Perinatal Data Collection (PDC) which records all births in NSW from public and private maternity service providers, including homebirths [1]. Women were included if they were at low risk of complications, that is:

- were 37 to 41 completed weeks of pregnancy
- had a singleton pregnancy in the cephalic presentation
- had no known medical or pregnancy complications (low-risk) including previous caesarean section and breech presentation
- had a spontaneous onset of labour
- Aged between 17 and 40 (inclusive)

Given that this study aimed to examine the trajectories of women who planned to give birth in the three available settings in NSW, we classified the women according to planned place of birth as recorded in the PDC. This dataset was obtained for the Birthplace in Australia Study, a national data linkage study of maternal and perinatal outcomes by place of birth (home, birth centre or hospital) [12]. A detailed description of the methods for selecting the women included in this study is described in Cheah et al. [30]. Briefly, women were excluded if they had any identified pregnancy complication (Table 2). For the remainder who laboured spontaneously between 37 and 41 completed weeks, we assumed their place of birth at the onset of labour was as planned.

**Table 2:** Complications in pregnancy: Variables used to exclude high-risk pregnancy

Dataset	Variables
Perinatal Data Collection	Maternal diabetes mellitus (pre-existing) Gestational diabetes Chronic hypertension Pregnancy-induced hypertension Pregnancy-induced hypertension – proteinuric Pregnancy-induced hypertension – non-proteinuric Any obstetric complication Breech or non-vertex presentation Born before arrival Received no antenatal care Previous caesarean section
Admitted Patient Data Collection (ICD-10-AM Codes)	Pre-eclampsia: O14 Eclampsia: O15 Chronic hypertension: O10, O11 Gestational hypertension: O13 Diabetes in pregnancy: O24 Prolonged rupture of membranes O42 Antepartum haemorrhage: O46 Maternal care for intrauterine death: O36.4 Vaginal delivery after caesarean: O75.7 Infants of women who were recorded to have a congenital abnormality (any Q code) were also excluded.

We stratified the decision tree by parity to investigate the impact and events related to planned birth settings, as the demographic details are significantly different for nulliparous women compared to multiparous women. Women who have an unplanned homebirth (born before arrival (BBA)) and those who free-birthed (that is, gave birth without a registered health provider present) were not included in this cohort. The age range indicated here corresponds with the age range categorised as ‘A’ in the Australian College of Midwives Consultation and Referral Guidelines [34]. Category ‘A’ refers to women at low risk of complications who fall under the scope of practice of a midwife. If a variance occurs, the Guidelines recommend the midwife consult either another midwife, a medical practitioner or refer the women to be overseen by a medical practitioner for secondary or tertiary care, depending on the significance of the variance.

### Data management and analysis

Data were received and analysed in SPSS V24. Groups were established according to the women’s intended place of birth as recorded in the PDC. The trajectories were determined using descriptive statistics to map the events that occurred throughout the labour, birth and postnatal period. These events represent the intended place of birth at the onset of labour, transfer to hospital (in labour or post-partum), mode of birth, and neonatal events including admission to special care nursery and neonatal intensive care. Data indicating mode of birth were missing in both nulliparous (120 cases) and multiparous (110 cases) hospital groups, therefore these cases were not included in the trajectories. Demographic data were stratified by parity; we used Chi Square test to compare grouped categorical data and univariate general linear model analysis of variance (ANOVA) to determine the differences in the means.

When allocating women who “transferred to HLW” from a BC for the decision tree, interventions such as epidural analgesia and instrumental birth were taken into account as some women who were recorded in the PDC to have given birth in a birth centre had received one or more of these interventions. These women were considered to have had a planned birth centre birth but were transferred to a hospital labour ward. Given these rooms are commonly adjacent to or near the labour ward for an alongside BC, the ‘transfer’ is assumed in this analysis. Freestanding BCs in NSW are not located near obstetric and neonatal services and as such, these women would have physically changed location. The proportion calculated in each branch are conditional on the number in the previous event (to the immediate left), adding up to 100%.

Neonatal transfer to higher-level care is reported in the NSW PDC as admission to Neonatal Intensive Care Unit (NICU) OR Special Care Nursery (SCN). Given the levels of care differ significantly in these two areas, this provides a crude measure of neonatal outcome. We calculated the length of stay of these babies and identified those who stayed in the NICU/SCN for greater than 48 h as a measure of more serious morbidity. Cases of intrapartum stillbirth and early neonatal death were retained in the trajectories (stillbirth was retained in the group who stayed with their mother and early neonatal death in the admission to NICU group). These numbers were very small (often *n* < 5, which meant they could not be reported due to ethical restraints regarding potential identification) and did not alter the conditional probabilities of the corresponding trajectory.

## Results

### Planned place of birth

A total of 496,387 women were included in the decision tree. The majority of women (464,630 93.6%) had their intended place of birth recorded as hospital, 29,951 (6.0%) intended a birth centre birth and 1824 (0.4%) intended a homebirth. There were differences in the demographic characteristics of the three groups with women intending a homebirth being older (32 years; standard deviation (SD) 4.7) than those in the birth centre group (30 years; SD 5.1) and hospital group (29; SD 5.3). A higher proportion of women in the hospital group were giving birth to their first baby (nulliparous) (45.1%) compared to the birth centre and homebirth groups (42.7 and 29.9% respectively) and the highest proportion of women with a gestational age of 40 weeks and over were women in the homebirth group (67.1%) compared with women in the birth centre (59.1%) and the hospital group (54%) (Table 3).

**Table 3:** Demographic characteristics by parity

Nulliparous women	Hospital *n* = 209,664 (%)	Birth Centre *n* = 12,782 (%)	Home *n* = 546 (%)
Maternal age (Years) Mean (SD)	27.5 (5.3)	28.34 (5.1)	29.8 (4.9)
< 20	17,018 (8.1)	645 (5.0)	14 (2.6)
20–24	45,614 (21.8)	2326 (18.2)	67 (12.3)
25–29	68,568 (32.7)	4351 (34.0)	177 (32.4)
30–34	57,497 (27.4)	3897 (30.5)	192 (35.2)
35–39	19,674 (9.4)	1485 (11.6)	89 (16.3)
40	1293 (0.6)	78 (0.6)	7 (1.3)
Previous pregnancies (≥20 weeks)			
0	209,664 (94.0)	12,782 (5.8)	546 (0.24)
Gestation (completed weeks) Mean (SD)	39.5 (1.06)	39.6 (1.07)	39.7 (1.1)
37	10,368 (4.9)	517 (4.0)	31 (5.7)
38	26,801 (12.8)	1405 (11.0)	51 (9.3)
39	56,144 (26.8)	3240 (25.4)	105 (19.2)
40	83,536 (39.8)	4768 (37.3)	229 (41.9)
41	32,815 (15.7)	2852 (23.1)	130 (23.8)
Multiparous women	Hospital *n* = 254,966 (%)	Birth Centre *n* = 17,151 (%)	Home *n* = 1278 (%)
Maternal age (Years) Mean (SD)	30.5 (5.0)	30.7 (4.8)	32.46 (4.3)
< 20	3715 (1.5)	121 (0.7)	3 (0.2)
20–24	35,569 (14.0)	1861 (10.9)	51 (4.0)
25–29	73,593 (28.9)	4753 (27.7)	262 (20.5)
30–34	90,026 (35.3)	6376 (37.2)	508 (39.7)
35–39	48,420 (19.0)	3767 (22.0)	415 (32.5)
40	3643 (1.4)	273 (1.6)	39 (3.1)
Previous pregnancies (≥20 weeks)			
1	150,364 (59.0)	10,727 (62.5)	662 (51.8)
2	65,633 (25.7)	4460 (26.0)	373 (29.2)
≥ 3	38,969 (15.3)	1964 (11.5)	243 (19.0)
Gestation (completed weeks) Mean (SD)	39.4 (1.03)	39.6 (1.02)	39.8 (0.98)
37	12,150 (4.8)	558 (3.3)	35 (2.7)
38	35,365 (13.9)	1828 (10.7)	112 (8.8)
39	72,906 (28.6)	4687 (27.3)	265 (20.7)
40	101,639 (39.9)	6789 (39.6)	592 (46.3)
41	32,906 (12.9)	3289 (19.2)	274 (21.4)

Note: Chi-Square Test was used to compare groups as follows: HB/BC, HB/Hospital, BC/Hospital in all categorical data. Results yielded statistically significant differences with *p* < 0.001 for all categories except gestational age (weeks) between BC and Home (*p* < 0.003). GLM also yielded significant differences at *p* < 0.001 between means in the above pairwise comparisons

**Fig. 2:**
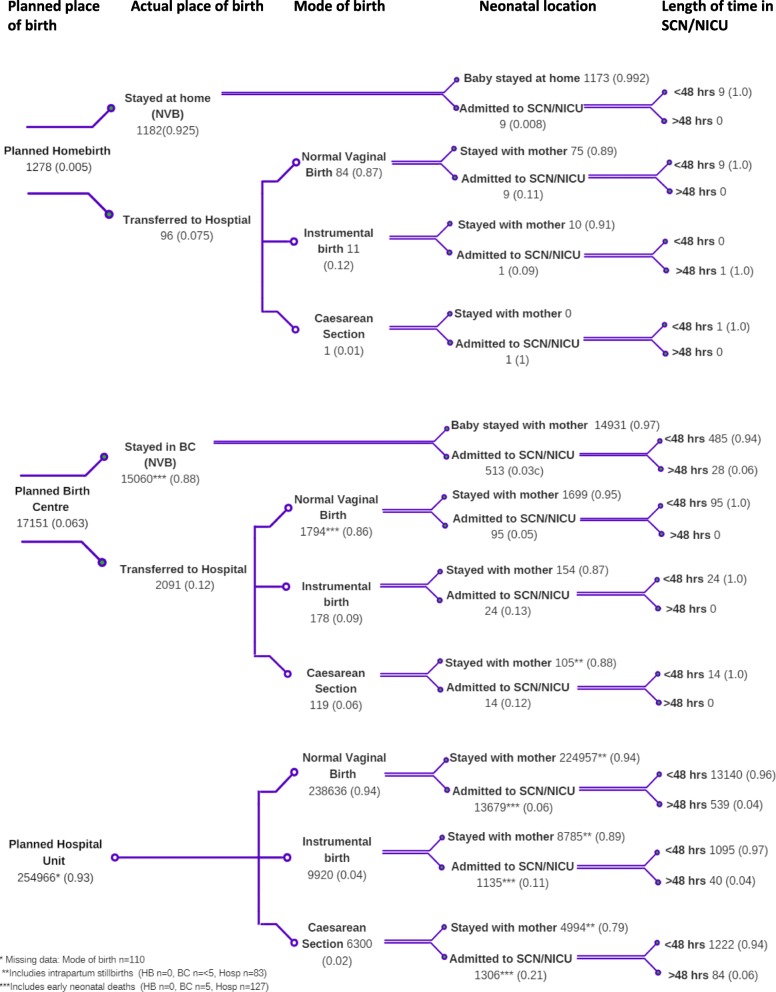
Multiparous women at low risk of complications between 2000 and 2012 (*n* = 273,395)

Figures 1 and 2 depict the decision tree constructed for this study. The decision node is the planned place of birth, separately for nulliparous and multiparous women. The trajectories the women take from the start of labour are represented by the ‘branches’ which emanate from the chance nodes named at the top of the figure: actual place of birth, mode of birth, neonatal location and length of time in special care nursery/ neonatal intensive care unit (SCN/NICU). Each branch extending from a chance node is given a probability of that event occurring.

### Nulliparous women

Of the nulliparous women, 0.2% planned to give birth at home, 5.7% planned a birth centre birth and 94% planned to give birth in a hospital labour ward. Of the women planning a homebirth in this group, 77.0% remained at home and had a normal vaginal birth (NVB). Of the 23% of women who transferred to hospital during labour, more than half (55.0%) went on to have an NVB. The rates of instrumental birth and caesarean section for nulliparous women planning a homebirth who were transferred to hospital were 22 and 23% respectively (see Fig. 1). These rates are 5.1 and 5.3% respectively when all nulliparous women planning a homebirth are taken into account. Of the women planning a BC birth, 66% remained in the BC and had an NVB. Forty-six percent of the women who transferred to the hospital labour ward had an NVB. The NVB rate for women in the planned hospital group was 62%. Of the women who transferred to the hospital from a BC, the rates of instrumental birth and caesarean section were 37 and 17% respectively. Overall, women in the planned BC group had lower rates of instrumental birth and CS compared with those in the planned hospital group (12.5 and 6.1% versus 23 and 15% respectively).

### Multiparous women

Multiparous women planning a homebirth had a 92.5% rate of NVB compared with 88.0% in a BC and 93.6% in the planned hospital group. Even following transfer, over 88% of women planning a homebirth had an NVB in hospital. In total, the vaginal birth rate in the multiparous birth centre group was 98.3%. Instrumental birth and CS rates were in the planned homebirth group were 12 and 1% respectively, following transfer (see Fig. 2).

### Neonatal trajectories

Infants of nulliparous women had higher rates of admission to NICU/SCN than multiparous women, with the largest proportion originating from the women who planned a hospital birth (10.1%). The smallest proportion of neonates admitted to SCN/NICU were admitted following a homebirth (1.7%). Of the planned BC group overall, 7.1% of neonates were admitted to the SCN/NICU. Infants of women who were transferred from home to a hospital in labour had a 16.7% NICU/SCN admission rate however as a proportion of all planned homebirths, the overall SCN/NICU admission rate was 5.1%.

Overall, fewer infants of multiparous women were admitted to the SCN/NICU with total SCN/NICU admission rates as follows: planned homebirth 1.6%, planned BC birth 3.6% and planned hospital birth 6.3%. The highest proportion of infants of multiparous women who were admitted to SCN/NICU were in the planned hospital group, following a CS birth (21%).

## Discussion

This study has used a decision tree framework to map the trajectories of women at low risk of complications planning birth at home, in a birth centre and in a hospital labour ward. Whilst there are options of birth setting for some women in NSW, the options do not meet demand. Women who would like to give birth at home are required to pay a private provider in the most part, and anecdotally, reports of waiting lists for birth centre care are common. This study aimed to illustrate the trajectories of healthy, low risk women to provide evidence on the rates of transfer and intervention in this group. This information is important to assist in planning of birthing services, and can also be used to inform estimates of costs of different places of birth. Overall, a greater proportion of women who planned a homebirth remained at home and had an NVB, followed by women who planned birth in a BC regardless of parity. Women choosing to give birth in a hospital received a higher level of intervention in both parity categories. Nulliparous women in both the homebirth and BC groups had higher transfer rates than their multiparous counterparts, however they had higher normal birth rates than the planned hospital group. These results demonstrate similar trends in NVB and instrumental birth rates for women at low risk of complications to international studies of place of birth [4, 6].

Transfer rates were lower compared to international evidence in both parity groups, particularly in the homebirth group. This could be attributed to a number of factors including the small number of women choosing a homebirth and careful planning and screening by the midwives who care for these women. In NSW, the majority of women who choose to give birth at home do so under the care of a midwife in private practise (MPP) which also requires personal funding, however there are a small number of publicly funded homebirth programs. The option of homebirth needs to be researched by the individual woman and extra effort required to find and engage a midwife who provides homebirth care. Women who choose a homebirth have confidence in the physiology of labour and birth, aspiration for a deeper relationship with her caregiver and a desire to be in a safe and familiar environment [35–37].

For women who planned birth at home or in a BC, those who required any intervention, including an epidural block or instrumental birth, were transferred to the hospital as these interventions are beyond the scope of care delivered in a BC. The majority of women who choose a BC used facilities that were within or adjacent to a hospital, as freestanding BC births account for around 15% of BC births during this time. This close proximity to medical intervention may influence the woman’s and the midwife’s ‘threshold for intervention’. However, a study in Sweden of adjacent birth centre facilities governed by the same hospital guidelines found that women had lower rates of intervention than their hospital labour ward counterparts [38], as seen in our study, however these proportions were higher than the homebirth group. Davis and Homer [39] investigated the impact of birth place on midwives in Australia and the United Kingdom and found that cultural influences, ie. adherence to policy, medical supervision and general environment influenced their delivery of care to women, particularly in the hospital environment.

Considering the women included in this analysis had a spontaneous onset of labour, it is not surprising that a largest proportions of the admissions to SCN/NICU were following instrumental and caesarean births, which could be related to either the need for an expedited delivery or the admission was as a result of an injury incurred during the birth. Similar rates of admission to SCN/NICU were shown in international studies of place of birth including a lower rate in multiparous women and women who planned a homebirth [21, 40]. Very few newborns were transferred to hospital following a homebirth, however the numbers are too small to draw any firm conclusions.

### Strengths and limitations

While data linkage is a powerful means to examine perinatal outcomes at a population level, there are limitations to the granularity of the data making close investigation of specific events challenging. Transfer from a birth centre to hospital is a good example. This study intended to highlight the trajectories of healthy women who could be reasonably compared across the three birth settings. Transfer from one setting to another is sometimes not recorded in the PDC, particularly when a BC is located within a maternity unit. For this reason, we used interventions such as epidural block and instrumental birth to indicate a transfer from BC to hospital. The numbers of women choosing a homebirth in NSW is very small and the probabilities associated with each trajectory in this group are less certain. With the benefit of linking data from across health datasets from one state, we were able to develop a cohort of women with comparable observable characteristics. It is difficult, however, to account for the unmeasurable or unobserved characteristics of women which fundamentally influence their choice of place of birth. Cases of stillbirth and neonatal death have been retained within the corresponding trajectories as these eventualities contribute to the trajectory of the mother and baby. This framework forms the foundation of a future cost analysis of place of birth using Australian Refined Diagnosis Related Groups. Closer investigation of morbidity and mortality was not within the scope of this paper. However, these outcomes have been reported on a national level in the Birthplace in Australia Study [12].

## Conclusions

This study has depicted the birth trajectories for women at low risk of complication and addresses the assertion that birth planned at home or in a birth centre results in a high rate of transfer, therefore adding an element of complication to an already delicate process. We have shown that a large proportion of women who begin labour at home or in a birth centre, stay in their chosen setting and indeed, even when transferred, have a high rate of normal vaginal birth. It is possible that the higher rates of intervention in hospital labour wards, even in a very low-risk group of women, could be avoided if women were given a greater choice of birth setting. Given this is the first time the trajectories of women choosing a birth outside hospital has been mapped, this evidence will assist the planning, costing and expansion of models of care in NSW.

## Data Availability

The data that support the findings of this study are not available. It is a condition of the agreement between the Centre for Health Record Linkage (CHeReL) and the researchers that the dataset remain confidential. We are not permitted to make any part of the linked data available to any party outside those named on the research team who have been granted access.

## Abbreviations

ABS: Australian Bureau of Statistics
APDC: Admitted patient data collection
CHeReL: Centre for health record linkage
NICU: Neonatal intensive care unit
NSW: New South Wales
PDC: Perinatal data collection
RBDM: Registry of births, deaths and marriages
SCN: Special care nursery

## References

[1] Centre for Epidemiology and Evidence (2017). New South Wales mothers and babies 2016.

[2] Scarf V, Rossiter C, Vedam S, Dahlen HG, Ellwood D, Forster D, Foureur MJ, McLachlan H, Oats J, Sibbritt D (2018). Maternal and perinatal outcomes by planned place of birth among women with low-risk pregnancies in high-income countries: a systematic review and meta-analysis. Midwifery.

[3] Hodnett ED, Downe S, Walsh D. Alternative versus conventional institutional settings for birth. Cochrane Libr. 2012. 10.1002/14651858.CD000012.pub4 PMC7061256 22895914

[4] Birthplace in England Collaborative Group (2011). Perinatal and maternal outcomes by planned place of birth for healthy women with low risk pregnancies: the Birthplace in England national prospective cohort study. BMJ.

[5] De Jonge A, Van Der Goes B, Ravelli A, Amelink-Verburg M, Mol B, Nijhuis J, Gravenhorst JB, Buitendijk S (2009). Perinatal mortality and morbidity in a nationwide cohort of 529 688 low-risk planned home and hospital births. BJOG Int J Obstet Gynaecol.

[6] Hutton EK, Cappelletti A, Reitsma AH, Simioni J, Horne J, McGregor C, Ahmed RJ (2016). Outcomes associated with planned place of birth among women with low-risk pregnancies. CMAJ.

[7] Davies-Tuck ML, Wallace EM, Davey M-A, Veitch V, Oats J (2018). Planned private homebirth in Victoria 2000–2015: a retrospective cohort study of Victorian perinatal data. BMC Pregnancy Childbirth.

[8] Homer C, Davis G, Petocz P, Barclay L, Matha D, Chapman M (2000). Birth centre or labour ward? A comparison of the clinical outcomes of low-risk women in a NSW hospital. Aust J Adv Nurs.

[9] Homer CS, Thornton C, Scarf VL, Ellwood DA, Oats JJ, Foureur MJ, Sibbritt D, McLachlan HL, Forster DA, Dahlen HG (2014). Birthplace in New South Wales, Australia: an analysis of perinatal outcomes using routinely collected data. BMC Pregnancy Childbirth.

[10] Monk A, Tracy M, Foureur M, Grigg C, Tracy S (2014). Evaluating Midwifery Units (EMU): a prospective cohort study of freestanding midwifery units in New South Wales, Australia. BMJ Open.

[11] Tracy SK, Dahlen H, Caplice S, Laws P, Wang YA, Tracy MB, Sullivan E (2007). Birth centers in Australia: a national population-based study of perinatal mortality associated with giving birth in a birth center. Birth.

[12] Homer Caroline S E, Cheah Seong L, Rossiter Chris, Dahlen Hannah G, Ellwood David, Foureur Maralyn J, Forster Della A, McLachlan Helen L, Oats Jeremy J N, Sibbritt David, Thornton Charlene, Scarf Vanessa L (2019). Maternal and perinatal outcomes by planned place of birth in Australia 2000 – 2012: a linked population data study. BMJ Open.

[13] Dahlen H, Schmied V, Tracy S, Jackson M, Cummings J, Priddis H (2011). Home birth and the National Australian Maternity Services Review: too hot to handle?. Women Birth.

[14] McIntyre MJ, Francis K, Chapman Y (2011). National review of maternity services 2008: women influencing change. BMC Pregnancy Childbirth.

[15] Dahlen H, Jackson M, Schmied V, Tracy S, Priddis H (2011). Birth centres and the national maternity services review: response to consumer demand or compromise?. Women Birth.

[16] Australian Health Ministers’ Advisory Council (2011). National maternity services plan.

[17] Commonwealth of Australia (2009). Improving maternity services in Australia: the report of the maternity services review. Department of Health and Ageing.

[18] Fox D, Sheehan A, Homer C (2018). Birthplace in Australia: processes and interactions during the intrapartum transfer of women from planned homebirth to hospital. Midwifery.

[19] National Institute for Health and Care Excellence (2017). Intrapartum care for healthy women and babies. In. UK.

[20] Blix E, Kumle MH, Ingversen K, Huitfeldt AS, Hegaard HK, Ólafsdóttir ÓÁ, Øian P, Lindgren HJ, Aoeg S (2016). Transfers to hospital in planned home birth in four Nordic countries–a prospective cohort study. Acta Obstet Gynecol Scand.

[21] Halfdansdottir B, Smarason AK, Olafsdottir OA, Hildingsson I, Sveinsdottir H (2015). Outcome of planned home and hospital births among low-risk women in Iceland in 2005-2009: a retrospective cohort study. Birth.

[22] Dixon L, Prileszky G, Guillilan K, Miller S, Anderson J, JNZCoMJ. Place of birth and outcomes for a cohort of low risk women in New Zealand: a comparison with Birthplace England. N Z Coll Midwives J. 2014;(50).

[23] Laws PJ, Tracy SK, Sullivan EA (2010). Perinatal outcomes of women intending to give birth in birth centers in Australia. Birth.

[24] Overgaard C, Møller AM, Fenger-Grøn M, Knudsen LB, Sandall J (2011). Freestanding midwifery unit versus obstetric unit: a matched cohort study of outcomes in low-risk women. BMJ Open.

[25] Stapleton SR, Osborne C, Illuzzi J (2013). Outcomes of care in birth centers: demonstration of a durable model. J Midwifery Womens Health.

[26] Blix E, Huitfeldt AS, Øian P, Straume B, Kumle M (2012). Outcomes of planned home births and planned hospital births in low-risk women in Norway between 1990 and 2007: a retrospective cohort study. Sex Reprod Healthc.

[27] Rowe RE, Fitzpatrick R, Hollowell J, Kurinczuk JJ (2012). Transfers of women planning birth in midwifery units: data from the birthplace prospective cohort study. [Erratum appears in BJOG. 2013;120(6):790]. BJOG.

[28] Coxon K, Homer C, Bisits A, Sandall J, Bick D (2016). Reconceptualising risk in childbirth. Midwifery.

[29] Bisits A (2016). Risk in obstetrics–perspectives and reflections. Midwifery.

[30] Drummond MF, Sculpher MJ, Torrance GW, O'Brien BJ, Stoddart GL (2005). Methods for the economic evaluation of health care programmes.

[31] Philips Z, Bojke L, Sculpher M, Claxton K, Golder S (2006). Good practice guidelines for decision-analytic modelling in health technology assessment: a review and consolidation of quality assessment. Pharmacoeconomics.

[32] Catling-Paull C, Foureur MJ, Homer CS (2012). Publicly-funded homebirth models in Australia. Women Birth.

[33] Coddington R, Catling C, Homer CS (2017). From hospital to home: Australian midwives’ experiences of transitioning into publicly-funded homebirth programs. Women Birth.

[34] Australian College of Midwives. National midwifery guidelines for consultation and referral. 3rd ed: Australian College of Midwives Incorporated; 2014.

[35] Burcher P, Gabriel J (2016). There is no place like home: why women are choosing home birth in the era of “homelike” hospitals. IJFAB.

[36] Borrelli SE, Walsh D, Spiby H (2017). First-time mothers’ choice of birthplace: influencing factors, expectations of the midwife’s role and perceived safety. J Adv Nurs.

[37] Coxon K, Sandall J, Fulop NJ (2014). To what extent are women free to choose where to give birth? How discourses of risk, blame and responsibility influence birth place decisions. Health Risk Soc.

[38] Gottvall K, Waldenstrom U, Tingstig C, Grunewald C (2011). In-hospital birth center with the same medical guidelines as standard care: a comparative study of obstetric interventions and outcomes. Birth.

[39] Davis DL, Homer CS (2016). Birthplace as the midwife’s work place: how does place of birth impact on midwives?. Women Birth.

[40] de Jonge A, Geerts CC, van der Goes BY, Mol BW, Buitendijk SE, Nijhuis JG (2015). Perinatal mortality and morbidity up to 28 days after birth among 743 070 low-risk planned home and hospital births: a cohort study based on three merged national perinatal databases. BJOG Int J Obstet Gynaecol.

